# Mapping of Industrial IoT to IEC 62443 Standards

**DOI:** 10.3390/s25030728

**Published:** 2025-01-25

**Authors:** Ivan Cindrić, Marko Jurčević, Tamara Hadjina

**Affiliations:** 1 University of Zagreb Faculty of Electrical Engineering and Computing, 10000 Zagreb, Croatia; ivan.cindric@fer.unizg.hr; 2 Končar—Digital Ltd., 10000 Zagreb, Croatia

**Keywords:** ISA/IEC 62443, Industrial Internet of Things (IIoT), cybersecurity standards, standards compliance, lifecycle management, zone segmentation, risk assessment, secure industrial environment, cybersecurity requirements, Operational Technology (OT)

## Abstract

The increasing adoption of the Industrial Internet of Things (IIoT) has led to significant improvements in operational efficiency but has also brought new challenges for cybersecurity. To address these challenges, a number of standards have been introduced over the years. One of the best-known series of standards for this purpose is ISA/IEC 62443. This paper examines the applicability of the ISA/IEC 62443 series of standards, traditionally used for securing industrial automation and control systems, to the IIoT environment. For each requirement described in the ISA/IEC 62443 standards, relevant research on that subject is reviewed and presented in a table-like manner. Based on this table, areas for future research are identified, including system hardening, asset inventory, safety instrumented system isolation, risk assessment methodologies, change management systems, data storage security, and incident response procedures. The focus on future improvement is performed for the area of system hardening, for which research and guidelines already exist but not for the specific area of IIoT environments.

## 1. Introduction

Over the past ten years, interest in the use of the IIoT (Industrial Internet of Things) has increased in various sectors such as manufacturing, production, energy, entertainment, transportation, healthcare, and building management. The Industrial Internet of Things is on such an upward trend that it is estimated to affect 46% of the global economy in some way, including almost 100% of energy production and 44% of energy consumption. The IIoT market as a whole is expected to exceed USD one trillion within the next three to five years [1].

However, the widespread adoption of the IIoT also increases vulnerability to cyberattacks, as the integration of IIoT devices into industrial systems goes hand in hand with the increasing connectivity of devices in industrial systems. This connectivity and diversity of devices significantly increases the attack surface of an industrial system [2]. Although these devices are useful, they can present security issues. They have limited resources, such as limited battery life or insufficient computing power to perform complex actions required to encrypt or decrypt data. They may have zero-day vulnerabilities that are difficult to to patch once the devices are in use, and they may have no device authentication, poorly configured control mechanisms, no secure communication protocols, and vulnerable web interfaces [1,3,4].

Due to these large impacts and potential losses, and due to the fact that the IIoT is considered vulnerable, it is important to analyze its security aspects and consider how it can be secured.

One way to secure a system or a process is to implement applicable security standards.

As defined in the NIST (National Institute of Standards and Technology) book *Cyber Security Standards*, “A cybersecurity standard is a document that defines both the functional and security requirements for a product, system, process, or technology environment” [5].

Of course, the use of cybersecurity standards also has disadvantages. One of the biggest problems is the fact that designing, discussing, and adopting standards is a lengthy process, often taking months to years. This pace is too slow for the rapidly evolving ICT landscape. In addition, companies with a dominant market position often insist on their own proprietary standards and tend to ignore third-party standards, as their adoption could strengthen their competitors. This creates a dilemma for customers who have to navigate a multitude of available standards to find the best solution for their needs. Proprietary standards can also lead to vendor lock-in, making it difficult to switch to a different vendor. It is also possible that one standard is not enough, so multiple standards need to be combined, increasing the complexity of implementation. On the other hand, standards can overlap or contradict each other [6].

Compliance with these standards is a resource-intensive effort and requires considerable time and financial investment on the part of companies. There is a risk of complacency once the standards are implemented. Companies may rely too much on compliance with the standards instead of actively managing security risks [3].

Finally, even a combination of several standards may not be able to fully meet the specific needs of the industry [7].

Nevertheless, a structured approach to securing a system using standards offers numerous advantages. First and foremost, standards improve consistency between manufacturers. This reduces variations which in turn enables better interoperability of products and processes. The standardization of technology and processes also reduces development costs. By using best practices and well-defined security requirements, standards provide a way to objectively compare products and measure their security features. Lastly, by participating in the implementation and development of such standards, the security community and companies share effort, knowledge, and best practices, resulting in a reduced effort required to develop solutions independently [5].

One series of standards that has been used in industrial environments for more than a decade is the ISA/IEC (International Society of Automation/International Electrotechnical Commission) 62443 series of standards. It defines requirements and processes for implementing and maintaining the cybersecurity of industrial automation and control systems (IACSs) and critical infrastructure [8].

This paper aims to align the requirements of Industrial IoT devices with the requirements of the ISA/IEC 62443 document series. To this end, the paper is structured as follows:

- The seconds chapter describes basic security requirements, and the differences in the importance of these requirements for IoT, IT, OT, and IIoT environments are presented. This is performed to provide a better understanding of the problems involved in mapping IIoT requirements to ISA/IEC 62443, which is more OT-focused.
- In the third chapter, the ISA/IEC 62443 series of standards is defined in more detail and the individual documents are described.
- Once the IIoT requirements and ISA/IEC 62443 are understood, the fourth chapter discusses previous work on this topic.
- Chapter five presents the ISA/IEC 62443 requirements, and for each requirement, it presents research conducted on that subject.
- Chapter six outlines opportunities for future research, and the lack of hardening processes in the context of the IIoT is analyzed.
- Finally, the Conclusions summarize the results, present the author’s opinion, and discuss the usefulness of the mapping provided by this paper.

## 2. Security Requirements’ Comparison for IIoT, IoT, OT, and IT

To better adapt the ISA/IEC 62443 documents to the IIoT environment, it is first necessary to identify the specific security issues related to the IIoT and how these specific security issues differ from the security issues in other environments, namely IT (Information Technology), the IoT (Internet of Things), and OT (Operational Technology). To accomplish this, the CIA triad (confidentiality, integrity, availability) is used to determine the primary security objectives for each of these environments. The CIA triad consists of three basic security requirements against which a system can be assessed: confidentiality, integrity, and availability [9].

Table 1 provides an overview of how these different environments—the IIoT, the IoT, OT, and IT—meet the security requirements of the CIA triad.

The following statements can be summarized from the table:

- For IIoT environments, availability is the most important requirement, followed by data integrity and finally data confidentiality.
- For IoT environments, integrity is most important, followed by availability and finally confidentiality.
- For OT environments, availability and data integrity are most important, and confidentiality is third by default.
- For IT environments, data confidentiality is the most important requirement, followed by integrity and availability.

Based on this table, it can be assumed that for both IIoT and OT environments, the priority order of the CIA requirements is as follows: availability is the most important, closely followed by integrity and finally data confidentiality.

The main reason for this prioritization is that for both IIoT and OT systems, great importance is placed on business continuity. This is because degraded availability has a direct and quantifiable economic impact and in many environments is also directly related to safety.

Integrity is also of great importance as it ensures the correctness and reliability of data. This is essential for maintaining safe and efficient operations. Any compromise to data integrity can lead to incorrect decisions and potentially dangerous situations.

Although data confidentiality is important, in IIoT and OT environments, it is considered less critical compared to availability and integrity. This is because the immediate risks associated with data breaches are often outweighed by the need for the continuous and accurate operation of systems. Therefore, maintaining uptime and data accuracy has traditionally taken precedence over data confidentiality [10,11].

The IIoT can be seen as a convergence of IT and OT [4,12]. The formal definition which can be found on the NIST website is as follows:

*“The sensors, instruments, machines, and other devices that are networked together and use Internet connectivity to enhance industrial and manufacturing business processes and applications.”*

[13]

OT can be formally defined as

*“A broad range of programmable systems and devices that interact with the physical environment or manage devices that interact with the physical environment. These systems and devices detect or cause a direct change through the monitoring and/or control of devices, processes, and events. Examples include industrial control systems, building automation systems, transportation systems, physical access control systems, physical environment monitoring systems, and physical environment measurement systems.”*

[14]

From both the security requirements in the table and the definitions above, it can be deduced that there is a certain overlap between the IIoT and OT. Since both technologies can coexist in the same environment and/or process, their security requirements and the way to protect them are also similar. This paper examines how similar these environments are and how exactly they differ through a detailed analysis of the applicability of ISA/IEC 62443 in an IIoT environment. Based on this, gaps in the security requirements are discussed.

**Figure 1:**
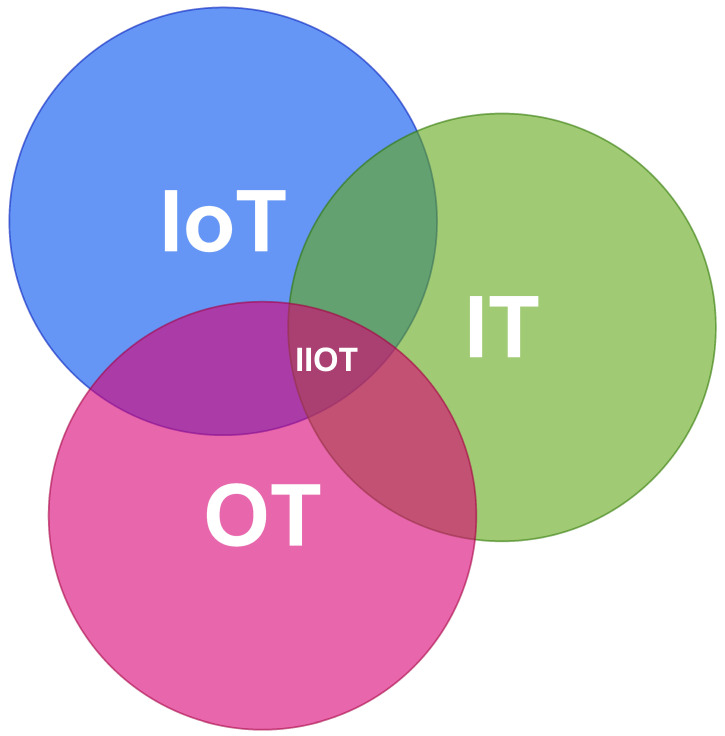
Venn diagram showing relations between IoT, IT, OT, and IIoT.

To further clarify the relations between the IIoT, the IoT, OT, and IT, Figure 1 is provided. From the table above, it can be concluded that the IIoT is an overlap of the IoT, OT, and IT. The IIoT uses the connectivity and data collection capabilities of an IoT environment but in an industrial, OT environment. And, there has long been a convergence between OT and IT in the sense that more and more IT technologies are being used in OT environments, which also applies to the IIoT. All in all, this means that there is a certain overlap between IT, the IoT, and OT, and this overlap can be referred to as the IIoT [11].

**Table 1:** Security requirements triad comparison for IIoT, IoT, OT, and IT.

Requirement	IIOT	IOT	OT	IT
Confidentiality	IT/OT integration introduces new vulnerabilities that did not previously exist, making it essential to implement robust confidentiality measures to prevent data breaches [2]. Maintaining confidentiality is difficult in Industrial IoT environments because devices do not have the computing power and security features required for adequate confidentiality [15].	The cryptographic algorithms used in IOT environments must be lightweight. Generic encryption cannot be used, and there is a need for fast algorithms [16,17].	Confidentiality is important when the system contains proprietary data, personal information, and critical operational data [18]. However, it is considered the least important of the three, especially when the data in question are only sensor data that are processed by the SCADA (Supervisory Control and Data Acquisition) system [19].	Confidentiality and integrity are paramount, while short-term downtimes do not pose a major risk [20]. Unauthorized access to data may lead to competitive disadvantages and even official sanctions [18].
Integrity	Any uncontrolled or deliberate modification of data may lead to dangerous incidents. The consequences can have a greater impact on safety, reliability, and resilience than those in a conventional IT system [2]. There are several vulnerabilities in wireless communication protocols. Therefore, data traffic must be continuously monitored to detect and mitigate threats [4].	In the absence of strict access control, tamper resistance, and strong authentication mechanisms, the integrity of data can easily be compromised [17]. Integrity is important because any manipulation of data can lead to wrong actions being taken based on those corrupted data [21].	Data integrity and data availability go hand in hand as the most important requirements in an OT environment [20]. Safe and reliable operation depends on accurate and untampered data. Data corruption could lead to unsafe conditions or incorrect decisions [18].	In a typical IT system, the primary concerns are data integrity and confidentiality [20]. IT focuses largely on the confidentiality and integrity of applications, services, and supporting technologies, while availability is by default in third place [3].
Availability	The Industrial IOT can be considered a subset of OT that has strict application requirements for continuous real-time operation and safety [1]. They are critical requirements as Industrial IoT devices manage real-time operations and critical infrastructure downtime results in financial losses [2].	IoT devices generally connect via wireless communication protocols such as Bluetooth, 802.11 (Wi-Fi), WiMAX (Worldwide Interoperability for Microwave Access), Zigbee, and others. The exploitation of these protocols has become a security concern in recent years [16]. The Internet of Things relies on continuous data collection and processing. Downtime can lead to interruptions and operational inefficiencies [17].	High availability is a critical requirement that requires, among other things, thorough pre-deployment testing as well as planned testing [20]. Downtime leads to financial losses and can even pose a safety risk [18].	Availability deficiencies may often be tolerated, depending on the specific use of the system [16].

Another metric of comparison is how these environments handle cyber-incident prevention, malicious behavior detection, and incident mitigation.

When it comes to prevention in IT environments, they rely on ISO 27001, an established standard for creating an information security management system in an IT environment [3]. The IoT relies on the ISO/IEC 27400:2022 standard, which provides guidelines on risks, principles, and controls for security and privacy in the Internet of Things [22]. OT is based on IEC 62443, which defines requirements and processes for implementing and maintaining the cybersecurity of industrial automation and control systems (IACSs) and critical infrastructure [8]. However, there is no standard for the Industrial IoT that could be considered established and widely used.

Threat detection is usually performed using intrusion detection systems (IDSs). In addition, security information and event management systems (SIEMs) and endpoint detection and response systems (EDRs) can be used. Both technologies are part of a Security Operations Center (SOC), a process that involves people and technology to monitor potential threats in real time. The difference is that SIEMs are centralized logging and analysis platforms, while EDRs are used to track endpoints. Mitigation is performed using intrusion prevention systems and regular vulnerability assessments. Machine learning techniques are used to detect abnormal traffic within a network [4,17,20,21,23]. Based on this research, it can be concluded that the detection mechanisms are similar in all environments, but the concepts of using a dedicated SOC in combination with SIEMs and EDRs are more advanced in an IT environment.

Mitigation is accomplished by isolating compromised devices, using automated threat response mechanisms, updating firmware and software, encrypting communications, access control mechanisms, and updating systems based on lessons learned from incidents [4,16,17,20,21]. As with the previous point, it can be seen that the principles of mitigation are similar. However, when it comes to industrial environments, whether OT or the IIoT, additional care must be taken to ensure the stability and availability of the system even when remedial action is taken, as can be seen from the results of Table 1.

## 3. ISA/IEC 62443 Overview

ISA/IEC 62443 consists of standards and technical reports dealing with issues of the security robustness and resilience of industrial automation control systems (IACSs). Robustness can be defined as the ability of a system to operate under a range of cybersecurity disruptions, while resilience can be defined as the ability of a system to recover from an unexpected cybersecurity event.

The aim of this series is to improve the availability, integrity, and confidentiality of systems used in IACSs, whether in the development phase by suppliers, in secure integration by an integrator, or a secure environment setup by the asset owner. The series is intended to be broad enough to be applicable to all types of equipment and systems in all industries. The term IACSs as defined in ISA/IEC 62443-1-1 includes the following:

- Systems such as DCSs (Distributed Control Systems), PLCs (Programmable Logic Controllers), SCADA, networked electronic measuring devices, and monitoring and diagnostic systems as well as hardware and software that are used within the IACS;
- Associated internal, human, network, or machine interfaces used for control, safety, production operation, and functionalities for continuous, batch, discrete, and other processes [24].

**Figure 2:**
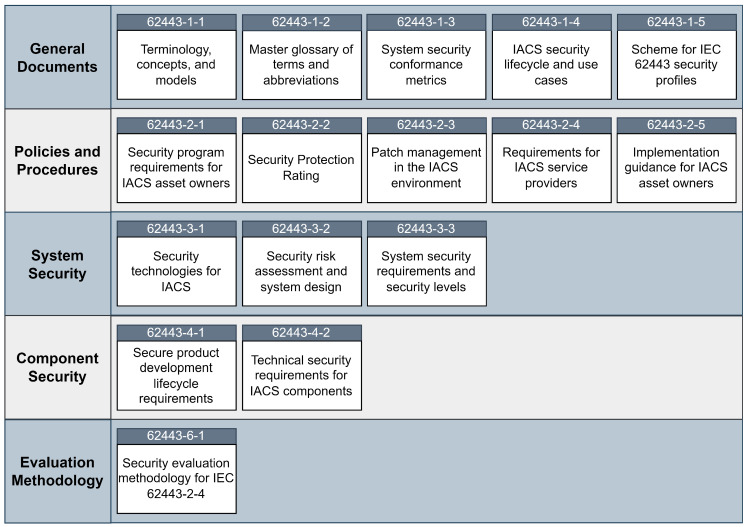
Division of IEC 62443 documents into five groups.

Figure 2 shows the documents in the series and their division into five categories: General Documents, Policies and Procedures, System Security Documents, Component Security Documents, and Evaluation Methodology.

The first four documents, found in the General Documents group, are informative in nature and provide the reader with a better understanding of the terminology, reasoning, and approach to security as defined by these documents. The terminology and concepts are formally defined so that the other documents in the series can be better understood. The metrics for security compliance are also defined in these documents. Using these metrics, it is possible to evaluate and improve the security of the assessed system [24,25,26,27].

The Policies and Procedures group contains documents whose primary purpose is to provide a framework for creating, maintaining, and improving security programs and policies and defining security controls. These documents provide a holistic approach to cybersecurity by establishing policies and controls for procedures throughout the system with the goal of mitigating potential risks. By following these documents, different organizations can achieve a consistent and standardized approach to cybersecurity and reduce variability by defining the controls outlined in these documents. The controls are defined by experience and best practices that have already been tested in the industry [28,29,30,31].

The System group focuses on the security of the IACS as a whole. It addresses the design, implementation, and management of system-wide security measures to ensure that the overall system is robust against cyber threats. The threats, mitigations, and risks are defined through a risk assessment, which ensures that the security measures are tailored to the specific threats and vulnerabilities of the system [32,33,34].

The Component group focuses on the security of the individual components of the IACS and not on the system as a whole. These documents cover the requirements and best practices for developing and managing secure components. Using these documents, components can be developed with security in mind, rather than security being an afterthought [35,36].

The Evaluation Methodology group provides security evaluation methodologies for the Policies and Procedures group. These documents are structured in a table-like format and provide conformance guidelines for security requirements. Using these documents, a system auditor can more easily determine whether the system conforms to the requirements specified in the Policies and Procedures group [37].

## 4. Related Work

At the time of writing, some research has been conducted on the use and applicability of the IEC 62443 series in the IIoT. However, none of these studies cover the entire IEC 62443 series or provide a comprehensive analysis of the improvements needed to make the standards more suitable for the IIoT. They mostly focus on specific parts of the standard such as risk assessment, threat modeling, or defense in depth. Furthermore, none of the papers address the applicability of these standards on a per-requirement basis but rather provide a general overview.

Hassani et al. [38] only focus on two parts of the IEC 62443 series, namely IEC 62443-3-2 and IEC 62443-4-2. They use these two standards to provide an example of a risk assessment model on a testbed using IEC 62443. Not all available standards are covered, nor do they directly question whether the standards are applicable to the IIoT. Furthermore, as the example is shown using a testbed, it is not general enough to be included as an addition to the standards that would make them more suitable for the IIoT.

Similarly to the previous paper, Schmittner et al. [39] mainly focus on only two standards: IEC 62443-4-1 and IEC 62443-4-2. They provide a defense-in-depth strategy developed based on these standards in combination with a threat model for an IIoT application. To achieve this, a threat analysis tool called ThreatGet is used. ThreatGet ensures comprehensive security by identifying potential risks and defining security measures for the entire lifecycle of the IIoT system. Again, this paper does not address the applicability of the standards and does not provide suggestions on how to make the standards more applicable to the IIoT.

Shaaban et al. [40] show how the concept of zones and conduits, normally used at the system level, can be applied at the component level to improve security. The study uses the MORETO (Model-based Security Requirement Management Tool) for security risk analysis, which follows the IEC 62443 guidelines to manage vulnerabilities in IoT components. The paper uses only this one concept from the IEC 62443 standard, without making any suggestions for improving the standard or the applicability of the IEC 62443 series as a whole.

Leander et al. [41] examine how the IEC 62443 standard can be applied to IIoT environments. The paper assesses the suitability of the standard for the IIoT and identifies areas where the standard may need to be updated to address the unique challenges of IIoT systems. It discusses the potential difficulties in maintaining security zone boundaries, communicating across these boundaries, and automating software updates. The analysis highlights the need for additional guidance and potentially new technologies to fully adapt IEC 62443 to the IIoT context. While the paper provides valuable insights, it does not offer a comprehensive overview of common IIoT vulnerabilities and their mapping to existing IEC 62443 requirements. Conversely, it also does not provide a complete mapping of the IEC 62443 series to an IIoT environment. Furthermore, given the rapid development of the IIoT and new versions of IEC 62443 standards, a more recent review of the results could be beneficial to ensure relevance.

Shaaban et al. [42] propose a novel algorithm to optimize the security measures for IoT devices in smart agriculture. This involves creating a threat model based on IEC 62443 standards. The aim is to ensure effective security mechanisms against potential cyber threats while minimizing redundancy and maintaining system functionality. The study uses the ThreatGet tool to analyze cyber risks and validate the optimized security attributes in an agricultural IoT application. The study focuses mainly on IEC 62443-3-3 and IEC 62443-4-2 and does not go into great detail on the applicability of the standards to the IIoT or whether they need to be modified in any way to be better suitable for the IIoT.

## 5. Mapping IEC 62443 Requirements onto IIoT

This chapter contains all the requirements that are included in all the available IEC 62443 documents.

The requirements are combined based on their thematic similarity and divided into subsections.

Each subsection contains an overview of the available research on that subject.

The requirements cover various aspects, such as asset management, cybersecurity program setup, risk assessment, audit methodologies, change management, segmentation, infrastructure, network security, defense in depth, wireless device security, safety instrumented system security and safety, physical security, data storage security, incident response procedures, business continuity procedures, backup, patching, training programs, personnel requirements and security, security policies and procedures, secure communication, authentication and identity management, media security, hardening procedures, secure development, cryptographic secret protection, testing, and decommissioning.

The requirements of IEC 62443-2-1 are used as the basis for the table and then expanded to include additional requirements from all other available parts of the IEC 62443 series.

IEC 62443-2-3 further extends the requirements for the patch management process [30].

IEC 62443-2-4 introduces new requirements not covered in previous documents and focuses on the role of service providers in the cybersecurity process [31].

IEC 62443-3-1 adds requirements and implementation guidance for technologies and systems that must be used to secure IACSs [32].

IEC 6243-3-2 also describes how to perform a risk assessment and how devices can be zoned according to use and type [33].

IEC 62443-3-3 contains system-wide security requirements that focus on technical security controls that apply to entire networks [34].

IEC 62443-4-1 defines how a software product can be protected throughout its lifecycle, from initial planning through to development and testing to continuous monitoring [35].

IEC 62443-4-2 lists similar requirements to the earlier documents but from the point of view of a single IACS component [36].

The purpose of this chapter is twofold. The first objective is to determine if there is sufficient research on each specific topic of the IEC 62443 requirement from the perspective of the IIoT. The second objective is to determine whether the IEC 62443 requirement is applicable to the IIoT. It may not be applicable if the requirement is too general to be applied to a specific environment or the requirement may not be applicable to the IIoT. The results of analyzing these two points are discussed in the next chapter as part of possible future directions for research on the application of IEC 62443 to an IIoT environment.

The research on the available papers was carried out using the following databases: Google Scholar, IEEE Explore, ScienceDirect, SpringerLink, ResearchGate, and MDPI. After the initial process of familiarization with the current state of research on this topic, the analysis of the available research was carried out as follows:

- All requirements from the IEC 62443 standards were compiled.
- The first part of the search query was always one of the following: IIoT, Industrial IoT, Industry 4.0, or Smart Factory.
- For each requirement, a number of combinations and synonyms were used to obtain a satisfactory number of articles on the topic of that requirement. For example, the search query for the requirement "Asset Inventory" contained one of the previous synonyms for the IIoT and one of the following: Asset Inventory, Lifecycle Management, Inventory, or Asset Classification.
- After performing this for all requirements, 107 documents were found.
- Those 107 papers were analyzed based on their keywords and their summaries to determine if they were related to the desired requirement. Of these 107 papers, 89 met the criteria.
- After a thorough review of these papers, 83 were deemed relevant to the desired area of work and these 83 papers are included in the following subsections.

### 5.1. Asset Inventory

An inventory of all assets, roles, and responsibilities for these assets should be created and maintained. The inventory should include the list of all current versions of software installed in the IACS. Roles and responsibilities for all assets should be created and maintained. Procedures for monitoring, alerting, adding, removing, and disposing assets must be established. All information must be linked to the appropriate information controls and defined retention periods. A regular review of the process and information should be carried out.

Lifecycle management processes and defined classification levels for information must be established for all information contained in the IACS. All data should be classified according to these levels.

The inventory should contain the list of all current versions of the software installed in the IACS.

Matsumoto et al. [43] propose an asset configuration management method that supports automation in diverse control system configurations and meets their specific requirements. Traditional asset management methods for conventional IT systems are difficult to implement for IIoT systems due to the variety of network protocols, devices, and technologies involved. Furthermore, these systems can be low-performing but must be guaranteed to function reliably in real time.

Kinnunen et al. [44] discuss the integration of the IIoT into asset management to ensure the relevance, accuracy, and timeliness of data. However, it is difficult to generate accurate and timely data for asset management as the complexity increases with more IIoT devices.

Bhanji et al. [45] emphasize the importance of connections within systems and note that current technologies cannot predict how a single device will affect the entire system. They recommend the use of advanced technologies such as machine learning to optimize asset management and analyze large data sets, as well as methods such as RCM (Reliability-Centered Maintenance), proactive/predictive maintenance, and lifecycle asset management to improve the asset management process.

### 5.2. Cybersecurity System

To begin the cybersecurity program, the top level of management must support it. The organization needs to have clearly defined responsibilities, and the team should be cross-functional to bring together the skills needed to ensure security in all parts of the IACS, whether internally or for external services and assets.

The scope of the cybersecurity management system (CSMS) must be defined. The scope should include all aspects of the IACS and integration points with business partners, customers, and suppliers.

Lackner et al. [46] note that a lack of professionals covering such a range of expertise is a challenge. Therefore, a security program must assemble a team that covers all topics, ranging from the IIoT, OT, cybersecurity, management, and all other aspects of the system and process.

Goto et al. [47] present a scenario in which they describe what a real-world example of IIoT/OT convergence would look like and what the process of adopting the IIoT into an existing environment should look like. The process includes executive support and the defining of roles and responsibilities covering all areas of the process. They propose a methodology for OT companies for the transition to Industry 4.0.

Mahmoodpour et al. [48] developed a dashboard that gives management better insight into the performance metrics of the system. Based on KPIs, different roles need to be defined for the dashboard.

Mugarza et al. [49] discuss the establishment of the overall cybersecurity management system based on IEC 62443, explaining that security risk management should be performed jointly by all entities involved in the system’s lifecycle. The real challenge arises from the fact that a large number of actors with different access rights and responsibilities are involved.

### 5.3. Risk Assessment

This involves selecting a risk assessment methodology based on threats and vulnerabilities specific to the IACS and providing all the information and resources necessary to conduct the assessment to the risk assessment participants. The chosen method of risk assessment and the results must be documented.

Firstly, a high-level risk assessment must be conducted and the scope should include risks to health, safety, and the environment (HSE). Secondly, a detailed vulnerability assessment must be carried out.

Once the assessment is completed, the frequency of reassessment and the criteria for reassessment must be defined. The risk assessment should be carried out at regular intervals, in all stages of a system’s lifecycle, including development, implementation, improvement, and decommissioning.

To address both physical and cybersecurity risks, a set of countermeasures must be defined.

The tools used for the risk analysis should be well defined, guidance should be provided, and recommendations for any negative impact of the tools should be available. The tools should be used in an agreed manner at a predetermined time.

Hassani et al. [38] note that the introduction of the IoT in industrial environments exposes industrial systems to traditional IT threats. Several risk analysis methods commonly used in regular IT systems have proven to be insufficient for IIoT environments. One of the biggest identified risks for this type of environment is the fact that IoT devices have no or limited authentication capability. The same applies to the communication capabilities of these devices. This type of insecure software can be compromised. To mitigate these problems, they suggest using the risk assessment method described in IEC 62443 and dividing the system into zones and conduits.

Schmittner et al. [39] suggest using the modeling tool ThreatGet to automate the threat analysis process. With the help of ThreatGet, the system can respond to the risks defined in the STRIDE model (Spoofing, Tampering, Repudiation, Information Disclosure, Denial of Service, Elevation of Privilege) and build an in-depth, layered defense based on them. They also note that IIoT devices have limited power, memory, disk space, and processing capabilities and that their introduction into the system increases the overall complexity of the system. Also emphasized is the fact that physical security is an important factor in IIoT environments.

According to Leander et al. [41], there is an obvious risk that parts of IEC 62443 may become obsolete due to the introduction of the IIoT. Security zones will be more difficult to maintain due to the dynamic nature of IIoT systems. The sheer number of devices in this type of system makes the conventional monitoring and updating procedures used in OT unfeasible. The potential lack of updates in turn makes the system vulnerable. They suggest classifying devices according to their criticality within the system and enabling automatic patch management for less critical components. To improve security, they also suggest the use of low-cost cryptographic methods, continuous monitoring, automatic vulnerability scanning, and software-defined networking (SDN) as a solution for managing dynamic connections and the use of micro-firewalls and the use of host configuration management (HCM) tools to manage all the diverse types of IIoT devices.

Nakamura and Ribeiro [50] propose a risk assessment that considers security, integrity, and reliability to calculate risks. The proposed risk assessment methodology consists of ten phases. The ten phases are determining the context, identifying points of attack, mapping threats, then mapping privacy, security, resilience, and reliability, then detecting vulnerabilities, estimating probabilities, estimating impact, multidimensional risk calculation, prioritizing security protocols, and finally determining an action plan. By considering both the physical and digital aspects of the system and the constantly shifting boundaries of the zones, the risks can be managed better.

Radanliev et al. [51] emphasize that supply-chain security should be considered in risk assessment. They explain that visualizing common risks in supply chains is crucial and that integrating real-time cognitive intelligence into risk analysis can help with assessments.

Tsiknas et al. [52] suggest that correlations and dependencies should also be considered in risk assessment. They suggest using artificial intelligence and machine learning for predictive cyber risk analysis to consider interdependencies and risks in the supply chain.

With the introduction of the IIoT, the traditionally compartmentalized OT environment is now exposed to multiple points of attack, as Zahran et al. [53] note. They propose IIoT-ARAS, an automated risk assessment system based on OCTAVE Allegro and ISO/IEC 27030 methodologies that can be used for IT/OT convergence and minimizing business disruption. The system performs regular audits, inventory checks, and predictive maintenance analysis.

Kandasamy et al. [54] warn that regular assessments of IoT systems have their limitations due to the drastic changes that can take place in such a system in a short period. Therefore, continuous assessment is required. The paper presents a risk ranking method to quantify IoT risks based on impact and the probability of occurrence. Risk assessment also needs to consider IoT-specific risks, e.g., cloud-related risks, real-time risks, autonomous risks, and risks related to recovery. The proposed solution is based on established risk assessment frameworks such as the NIST (National Institute of Standards and Technology), OCTAVE (Operationally Critical Threat, Asset, and Vulnerability Evaluation), the ISO (International Organization for Standardization/International Electrotechnical Commission), and TARA (Threat Analysis and Risk Assessment), adapted for IoT environments.

Regarding the lifecycle of machines, Kivelä et al. [55] say that safety and security should be assessed together and cyclically throughout a machine’s lifecycle based on FMVEA (Failure Mode, Vulnerabilities, and Effects Analysis) and STRIDE. As specified in the IEC 62443 standard, the safety-related parts of the system should be separated from the communication parts of the system in order to facilitate the maintenance of the machine, which is also confirmed by the authors.

Cyber-physical systems are becoming increasingly connected through the integration of more and more IIoT devices, and, therefore, the physical world can be affected by digital problems, as Lyu et al. [56] state. The authors propose an AI-driven, online, real-time risk and reliability assessment that continuously monitors the system and provides real-time feedback on potential threats and failures.

### 5.4. Audit Methodology

Audit methodology includes defining audit methods and metrics, conducting audits, and creating a document log. The storage space required for audit records should be determined according to commonly recognized recommendations for log management and system configuration. Log records should satisfy the security requirement of non-repudiation.

The system should be improved based on the knowledge gained from the audit trails.

Lackner et al. [46] propose the use of blockchain technology, which improves the security and integrity of document paths. To reduce the data load and response times due to the larger amount of data in an IIoT environment as opposed to a traditional OT environment, this storage requirement is met by edge computing. To audit the system, the authors suggest the use of a CSAM (Cybersecurity Audit Model). This is a system for assessing and validating audit, preventive, forensic, and detective controls that was developed to overcome the limitations of the existing cybersecurity audit process. The CSAM was proposed by Sabillon et al. [57].

To ensure data integrity, Fan et al. [58] developed Dredas, a decentralized, reliable, and efficient audit system for outsourced data. It utilizes blockchain, which allows anyone to audit the data, and is based on Ethereum contracts. Moreover, the proposed system is scalable, which makes it suitable for the IIoT. The result of the audit is made public so that everyone can benefit from it.

### 5.5. Change Management System

A change management system should be created and maintained for all IACS components. This includes a risk assessment of all changes and written security guidelines for all changes. The security requirements for all assets should be defined. The maintenance of all assets should be documented, tracked, and carried out promptly.

According to Sugandi et al. [59], there are a number of issues with implementing a change management system in an IIoT environment. Organizations tend to resist change, which makes timely maintenance and tracking more difficult than it should be. The lack of a strategic approach to change management can lead to partial implementations. IIoT environments are made up of different devices and systems, making a consistent change management system very complex. Successful change management requires thorough planning, stakeholder involvement, effective communication, and a proactive approach.

It is important to proactively anticipate changes required by an external context, as described by Farina [60].The process of change management is a systematic approach consisting of processes, tools, techniques, individuals, and external third parties. To create a functioning change management process, cross-functional teams are needed and iterative processes such as agile project management should be used. Training must be provided, collaborative tools used, and the decision-making process restructured. Big data analysis should be used to monitor and maintain the systems.

### 5.6. Defense in Depth

The defense in depth model should be used to implement multiple layers of defense with a risk-based approach based on the threat model.

The previously mentioned ThreatGet tool presented by Schmittner et al. [39] can be used for defense in depth by applying it to several aspects of security management, such as security requirement specification, secure-by-design principles, secure implementation processes, verification and testing, security issue management, update management, and security policy creation.

To create a defense-in-depth model, Wai and Lee [61] suggest the use of a multi-layered defense-in-depth (DID) framework. The framework combines various security practices such as access controls, network segmentation, anomaly detection, encryption, policies, processes, and technologies.

One of the biggest problems with applying security to OT systems is that they often contain legacy devices that either do not support security features such as authentication, encryption, and monitoring, do not have them, or are underpowered, according to Mosteiro-Sanchez et al. [62]. They suggest lightweight encryption protocols, the creation of secure zones in the network design so that the network is tailored to the IIoT, and the use of mechanisms such as Attribute-based Encryption (ABE), a type of encryption that relies on attributes that the entity possesses rather than an entity’s public key.

Ning and Jiang [63] talk about the use of defense in depth to limit access by potential insider threats. They propose a three-layered architecture consisting of intrusion detection systems, data-driven analysis to detect irregular patterns, and model-based techniques to detect anomalies in the behavior of a physical process. Once the attack is detected, defensive measures are also taken to ensure system security.

### 5.7. Segmenation

All components of the IACS should be identified and the devices should be grouped into logical segments. Simple network diagrams should be created for all components, showing devices, network types, and the physical and logical location of all devices. In addition, detailed logical and physical infrastructure drawings and documentation, including network devices, interfaces, system descriptions, guidelines, installation guides, and inventories, must be created, maintained, and updated.

To secure the system, procedures for network segmentation architecture based on zones and conduits, DMZs (demilitarized zones), network- and host-based firewalls, VLANs (Virtual Local Area Networks), intrusion detection systems (IDSs), and intrusion prevention systems (IPSs) and the blocking of unnecessary communication with blocking devices should be carried out. DHCPs (Dynamic Host Configuration Protocols) should be deactivated.

IACS devices should be grouped into zones that are logically or physically separated from business or enterprise systems. Custom operating systems used on the devices in an industrial environment should be isolated and the traffic generated by these devices should be separated from information traffic.

Bogaz Zarpelão et al. [64] warn that when implementing an IDS in an IIoT environment, one must consider the fact that the LLN (Low-Power and Lossy network) nodes have limited computational capabilities. Also, those nodes have to operate in real time, which means that some care must be taken to ensure these requirements. To cope with the problems of implementing such an IDS, the researchers propose a combination of signature-based, anomaly-based, and specification-based IDSs. One prominent solution, SVELTE (Secure Virtual Environment for LTE (Long-Term Evolution)), focuses on lightweight detection suitable for IoT nodes with limited resources, while more resource-intensive tasks are outsourced to centralized nodes.

To tackle the complexity of IIoT systems, Bogaz Zarpelão et al. [64] propose the use of machine learning-based intrusion detection systems designed to work with IIoT-specific devices and traffic. Extensive testing and large amounts of data are required to create such a system.

Although network segmentation offers benefits, it also presents challenges, especially in a complex IIoT environment. Baligodugula et al. [65] use several approaches to segment an IIoT environment: physical segmentation, virtual segmentation, firewall rules, network access control, and micro-segmentation. They also provide real-world examples of effective IIoT segmentation strategies.

Micro-segmentation restricts access within each segment of a network by imposing security rules that in turn prevent unauthorized communication between devices outside their boundaries. Since micro-segmentation has advantages but is a complicated process, Arifeen et al. [66] propose to automate it using the OPTICS (Ordering Points To Identify the Clustering Structure) clustering algorithm based on traffic patterns. A decision tree classifier is then used to classify network traffic within these segments as normal or malicious.

Nano-segmentation or per-device segmentation, called Hopper, is a solution proposed by De Vaere et al. [67]. It restricts the communication of each device to explicitly whitelisted data streams, preventing lateral movement and unauthorized access. The solution uses hierarchical keys to authenticate and verify each packet at each step in the network.

Romarick et al. [68] note that IIoT systems can be subdivided based on factors such as IP addresses, physical location, traffic intensity, or the protocols used. Firewalls and DMZs are used to create protection between critical areas. However, the authors warn that segmenting an IIoT network is not a trivial task. Although such segmentation is recommended by various standards, the actual implementation is only vaguely described, leaving much to the discretion of the implementer.

Ihirwe et al. [69] propose the use of CHESSIoT (Compositional and Predictable Assembly of Embedded Systems for Internet of Things), a modeling tool that supports the development and analysis of IIoT systems by breaking down complex systems into manageable components and specifying their interactions.

Boyes et al. [70] use the Purdue model to divide the IIoT into logical zones through the use of firewalls and conduits based on factors such as device location, connectivity, and functionality.

### 5.8. Wireless Devices

For all wireless devices, it is necessary to document security mechanisms that prevent unauthorized access and protection for remote access, including mechanisms for authentication and access control. All wireless devices must be authorized and monitored.

Wireless communication must be encrypted using cryptographic mechanisms that are widely recognized in both the security and industrial automation industries.

All restrictions set by system owners for the secure operation of wireless devices must be defined and enforced.

Wireless devices must be separated from wired devices.

The documents mentioned earlier state that the security of wireless devices can be improved through the use of SDN, encryption, intrusion detection systems, secure communication standards, and the separation of wireless devices from wired devices through zones and conduits [52,63,71,72,73].

To secure an industrial wireless sensor network (IWSN), Raposo et al. [74] propose a flexible, cross-standard monitoring architecture that minimizes the burden on sensor node resources and leverages already-available network metrics and widely used management standards. Specifically, the paper deals with the WirelessHART (Wireless Highway Addressable Remote Transducer), ISA100.11a, WIA-PA (Wireless Networks for Industrial Automation–Process Automation), and ZigBee, for which they claim there are known attacks. The authors propose ISA100.11a for monitoring and authorizing communication.

For authentication, Dhirani et al. [3] suggest the use of Kerberos, a widely used network authentication protocol. Other viable solutions include blockchain technologies and multi-factor systems such as PassLogic or cloud-based key management services (KMSs) such as the AWS (Amazon Web Services) KMS, Azure Vault, and Cryptomathic KMS.

Bahri et al. [72] warn that some existing technologies, including Zigbee and IEEE 802.15.4, have certain limitations when it comes to their applicability in IIoT environments and therefore suggest the use of technologies such as Low-Power Wide-Area Networks (LPWANs).

Mahmood et al. [73] talk about wireless technologies in the context of the IIoT. To secure wireless networks, they mention the Root of Trust (RoT), a mechanism that is used to authenticate a device before it can join a network and can be embedded in hardware. Furthermore, the use of standardized cryptographic algorithms is recommended, such as AESs (Advanced Encryption Standards) in combination with a hardware-based random number generator (RNG). Due to the dynamic nature of the IIoT environment, regular audits and updates of the network are required. One of the tools mentioned for this purpose is FOTA (firmware-over-the-air) updates.

### 5.9. Safety Instrumented Systems

Safety instrumented systems (SISs) should be logically and physically separated from the rest of the system to avoid any kind of propagation of malfunctions in both directions.

Remote access to the SIS should be disabled.

For physical separation, a dedicated chip or a dedicated gateway can be used to separate the security network from the rest of the network. Containers and hypervisors can be used for virtual separation, but solutions for IIoT environments are only slowly emerging [73].

Johnson [75] warns that disabling remote access, while important for security, compromises the operability of the system. To isolate parts of the system, the author suggests the use of data diodes, buffer networks, and VPN tunnels.

Onshus et al. [76] also say that, in general, the security system should be completely separated physically and logically. Furthermore, one should not only rely on separation but take several measures that follow the principle of defense in depth. Data diodes, security zones, and lossless protocols can be used. The Purdue model proposes the idea of logical separation between SISs and other network systems.

However, complete separation in a modern IIoT environment is not possible [76].

### 5.10. Physical Security

A physical security perimeter must be established to protect against unauthorized access, tampering, or intentional, unintentional, or environmental damage.

Abuserrieh and Alalfi [77] provide an overview of existing technologies and mechanisms for safety and protection. They suggest tools such as IoTSEER, HAWatcher (Home Automation Watcher), and Peeves (Physical Event Verification System) for dealing with environmental damage.

Yang et al. [78] categorize physical threats and discuss possible countermeasures. Tampering can be detected by solutions such as Physical Unclonable Functions (PUFs) or by detecting the removal or theft of power lines. The use of RFID (Radio Frequency Identification), infrared sensors, and video surveillance is recommended to detect and prevent physical access that could lead to detrimental consequences. GPS and other tracking technologies can be used to protect against theft. Biometric authentication can be used to protect against unauthorized access, and behavioral analysis, such as of a user’s movements or the way they type, can be used to detect unusual interactions with the system.

### 5.11. Data Storage and Data Transfer

Data storage points and data flows within the ICAS must be documented, including the security requirements for securing them (e.g., confidentiality, integrity).

Authorization, retention periods, cryptographic controls, access points, and deletion policies should be defined for the data flows.

All scripts, executables, and other important files should be protected by an integrity checking mechanism.

Abdulghani et al. [79] provide a framework for data at rest. They suggest restricting data access through authorization, setting minimum data retention periods, and encrypting data at rest. A clear end-of-life technique should be implemented for each IoT device so that any object of this type can be disposed of without exposing sensitive data. To ensure the integrity and confidentiality of data, both they and Wang et al. [80] suggest the use of blockchain-based technology.

To ensure the security of the data flow, Wei et al. [81] propose the use of mechanisms such as Information Flow Control (IFC), where the data sent are tagged with administrative flags to control who can access the data. They also mention some other technologies such as UCONABC (Usage Control Model with Authorizations, oBligations, and Conditions), which can change permissions in real time, and the use of network-level security monitoring.

Fu et al. [82] describe securing data in cloud and fog computing environments. They propose encrypting both operational data and serial numbers, with each node having a secret key. Data storage is managed by dynamically updating ID-AVL (Identity-based Adelson-Velsky and Landis) and RF (Random Forest) trees that enable efficient data searching. Fog computing is used for processing and storing sensitive data, while cloud computing is used for non-sensitive data. To protect and encrypt the data, they use the k-nearest neighbor (kNN) algorithm.

### 5.12. Resilience

The system should be able to operate in a degraded mode during an incident, such as an intentional attack, a denial-of-service attack, or other attacks that have been identified as a potential risk. All security breaches must be documented and communicated in order to learn lessons from the incidents. Incidents should be identified, reported, and dealt with in a timely manner.

In the event of a system failure, the recovery objectives and the impact and consequences associated with the failure of one or more systems must be defined. An incident response plan and procedure for reporting unusual activity must be established.

To deal with faults within a system, Zhou et al. [83] developed a fault-tolerant transmission mechanism for SDN-based IIoT fiber-optic networks. The mechanism works with parallel transmission in combination with sparse network coding. It can recover from node failures and cope with packet loss. By using SDN technology, it can dynamically adjust routing based on reliability, availability, and delay. Since most of the data sent and received in an IIoT environment are time-based measurement data, a reliable time synchronization mechanism is important.

Priyanka et al. [84] propose a fault-tolerant system for time synchronization based on packet-coupled oscillators (PkCOs).

Wu et al. [85] propose a multi-level strategy that includes self-protection for redundancy and reliability, self-configuration to maintain operability in the event of faults, and self-healing to restore normal operation after damage. By using flexible communication techniques, the system can reroute itself to avoid collapse. The authors also mention the importance of redundancy to increase the resilience of a system.

Lee and Chung [86] propose an automatic method of selecting backup nodes to minimize recovery time. The nodes are selected using regression analysis.

Cook et al. [87] focus on adopting traditional IT incident response frameworks and adapting them to an IIoT environment. This includes pre-incident preparation, incident detection, investigation, response strategies, and reporting.

### 5.13. Business Continuity

Business continuity plans and a business continuity team with clearly defined roles and responsibilities must be created.

Backup and recovery procedures should be established in line with the business continuity plan.

The business continuity plan and the backup and recovery procedures must be regularly tested and updated if necessary.

The most common risks identified by Ali et al. [88] in connection with the IIoT are unauthorized access to data and the disruption of services. The authors present a framework for business continuity that is aligned with the IIoT. To achieve this, they propose adhering to well-known standards such as ISO 22301.

To create a backup, Chang et al. [89] propose a cloud backup using the Apache Spark platform. This approach uses algorithms for clustering and mapping data so that data can be restored quickly and reliably.

### 5.14. Policies and Procedures

IACS cybersecurity and safety policies and procedures must be developed, maintained, reviewed, and regularly updated.

Kumar et al. [90] warn that the standardization of IIoT environments is not mature enough.

Wai and Lee [61], as previously mentioned, provide a defense-in-depth solution that includes the creation of policies and procedures for security, among other things.

Ali et al. [88] talk about ensuring business continuity by managing various aspects of business, but they mention that there is a lack of frameworks and guidelines when it comes to creating policies and procedures for the IIoT.

### 5.15. Patching Procedure

The patching procedure must include testing the patch before using it in a production environment.

Patching should be performed by authorized personnel within the allotted time. The patching procedure must also be performed in a secure manner to maintain product integrity.

The procedure, patch descriptions, possible consequences, and warnings should be documented.

All security measures must not impair the essential functions of the IACS or the availability of the IACS in any way.

Serror et al. [91] warn that there may be many long-lived legacy components and an increasing number of devices. They suggest automatic patch management, fine-grained access control, and network monitoring. To deal with obsolete components, they suggest intrusion detection systems.

According to Aman et al. [92], the testing of applied patches is crucial. The traditional approach of scheduled maintenance periods is slow and complex and is not fully suitable for a dynamic IIoT environment. The authors present IoT-Proctor, a lightweight framework designed to stop the spread of malware within a network through a structured patching process. The framework uses Physical Unclonable Functions (PUFs) for device identification and categorizes devices based on their security status.

Mugarza et al. [49] propose a structured patch lifecycle model for testing, approval, and installation that is compatible with IEC 62443. They outline the key responsibilities of device owners, product suppliers, and service providers in a patching process.

### 5.16. Personnel Training and Security

A cybersecurity training program should be developed for all employees. The training program must be validated at regular intervals and revised based on the results of the validation. Employee training records must be retained.

A security policy for staff must be drawn up. Depending on the severity of the system, personnel must be tested at the beginning and at regular intervals. A policy must be drawn up for all staff, setting out the security responsibilities of all staff and the physical security and cybersecurity required to protect staff.

Karampidis et al. [93] note that there is a shortage of technical staff in the labor market who are familiar with both cyber risks and operational requirements. Such training is expensive. To counteract this, the authors present the InCys 4.0 project, which is an open-source collection of training materials and courses.

Benis et al. [94] emphasize the need for continuous learning and updating the curriculum taught to students.

Fitsilis et al. [95] propose a competency framework based on industry requirements rather than a specific occupation.

Kopytko et al. [96] present a human resource management mechanism that includes planning, control, continuous analysis, and motivation systems to increase the efficiency, safety, and security of personnel and to improve the system.

### 5.17. Device-to-Device Security

Device-to-device authentication mechanisms should be implemented.

Authentic data sent by the sending device should be validated as authentic by the receiving device. All devices should have the ability to authenticate for network services. This means that networked devices should be able to differentiate between authorized and unauthorized remote requests for data or to perform actions.

Lara et al. [97] propose the use of the Lightweight Authentication and Key Distribution (LAKD) protocol, which was developed specifically for resource-constrained IIoT devices. It is based on lightweight operations such as xor, addition, subtraction, and hashing to enable authentication at a low computational cost. The protocol allows the endpoints of the communication to authenticate each other and validate the messages sent between them so that the messages cannot be forged, replayed, or altered, while the communication itself is not vulnerable to man-in-the-middle attacks.

To avoid having to rely on a third-party certificate authority, Shen et al. [98] use a blockchain-based authentication and key agreement mechanism called BASA (Blockchain-Assisted Secure Authentication). This allows devices to be authenticated with identity-based signatures (IBSs) and enables secure communication between these devices.

### 5.18. Identity, Authentication, and Account Management

Identity, authentication, and account management systems must created.

Users should only be authorized for access to required resources and only for a specific period of time, whether for local or remote connections. All connections must be defined so that the purpose of the connection, the type of connection (application and technology used), the location and identity of a person making a connection, and the duration and timing of the connection are known.

Authorizations should be reviewed regularly. Unnecessary accounts should be blocked or removed. Access records should be retained.

Password policies for regular updating and a minimum password strength must be enforced. Password management and distribution systems should be capable of managing and distributing passwords in a secure manner.

A session lockout mechanism should be defined and enforced.

Systems should be able to uniquely identify and authenticate all software processes and services, including their locations.

Alsaadoun [99] developed an identity-and-access management framework that is an extension of RAMI 4.0 (Reference Architectural Model for Industry 4.0). The framework addresses issues of user identity management, account provisioning and retirement, security credential handling, authorization release, the segregation of duties, role-based access control and role management, and data leakage. They emphasize the need for the traceability of actions and the non-repudiation of these actions.

Gouglidis et al. [100] analyzed the access control mechanisms in an IIoT environment and came to the conclusion that role-based access control (RBAC) is more suitable than attribute-based access control. They also suggest using edge, fog, and cloud layers for distribution, policy enforcement, and data processing. They emphasize the need for the dynamic separation of duties to help with session management.

To deal with the low power of typical IIoT devices, Mohaghegh and Ngo [101] propose physical layer authentication that is lightweight. It utilizes the physical characteristics of the devices and the characteristics of the transmitted signals. However, the authors point out that this approach has limitations due to factors such as signal noise.

To ease the load on end devices, Astorga et al. [102] developed a scalable identity management system for IIoT environments known as the Certificate Lifecycle Management (CLM) server, which is based on X.509 certificates. By using CLM, the complexity of certificate management is shifted to an edge server, while the private keys remain on the IIoT devices. By using this system, authorization, account flow, and authentication are ensured.

The framework, built on the ForgeRock Open Identity Stack from Preuveneers et al. [103], unifies the identities of users, cloud services, and devices to ensure that each of these entities can be authenticated, be authorized, and operate with appropriate permissions. The framework also provides an identity store to track entities within the system.

To deal with the resource constraints of IIoT devices, R. et al. [104] propose the use of multi-factor authentication that combines passwords, biometrics, and smart cards to identify users and devices.

### 5.19. Media Security

Defined access guidelines for handling media should be established for all media. All media should be labeled and stored, handled, and transported safely. When media is no longer in use, no longer needed, or defective, media should be cleaned and disposed of safely.

The EMSDE method (Enhanced Modern Symmetric Data Encryption) proposed by Suresh and Priyadarsini [105] secures IoT data by applying multiple layers of encryption. First, the data are encrypted as they are transmitted from IoT devices to the gateway system. Then, EMSDE adds another layer of encryption at the gateway before the data are transferred to the cloud.

The Fog Computing Security Storage Model (FCDSSM) proposed by He et al. [106] secures the storage of data in fog nodes. The architecture includes multi-level trusted domains to securely manage data in geographically distributed environments. The system uses key management and authentication mechanisms to ensure the confidentiality and integrity of the data.

### 5.20. Hardening Procedure

Policies and procedures for the hardening of IACS components should be established. Metrics on the success of the hardening process should be developed.

Hardening should define the processes that determine how unnecessary software and services, physical and logical access points, devices, communications, media, network addresses, unused ports, session locking, certificate and cryptographic key handling, network security, etc., are removed.

Laszka et al. [107] use a layered approach to system hardening that includes redundancy, diversity, secure coding, firewalls, and penetration testing. They also present a meta-heuristic algorithm to find near-optimal designs for IIoT systems because, as the authors say, complexity and cost must be considered in addition to security when designing a system.

To ensure the security and reliability of IIoT systems, Barekar et al. [108] use machine learning techniques to monitor network traffic, detect anomalies and intrusions, and predict system failures in real time. By continuously learning from past events, these models can adapt to new threats, creating a dynamic defense.

Bi et al. [109] evaluate defense strategies based on the total system loss and total cost of the defender. They also emphasize the need for the continuous updating of defense strategies. They explore strategies for defense against Advanced Persistent Threats (APTs) in IIoT systems. Their simulation of defense and attack is based on the Stackelberg game model.

Threat mitigation can be achieved through secure development practices by using authentication, secret management procedures, encrypted network communication, ACLs, regular updates, VPNs, and user awareness, according to Kumar et al. [90].

When it comes to intrusion detection in the IIoT, there are several types of IDS technologies that are suitable for the IIoT. These technologies include traditional IDS types such as signature-based IDSs, anomaly-based IDSs, specification-based IDSs, or hybrid IDSs. Signature-based IDSs detect threats using predefined signatures of known attacks. Anomaly-based IDSs detect deviations. Specification-based IDSs use manually defined rules. Hybrid IDSs are combinations of the aforementioned IDS technologies. Depending on the location of the IDSs, they can be divided into host-based IDSs, network-based IDSs, distributed IDSs, and centralized IDSs. Host-based IDSs monitor activities on a single device. Network-based IDSs monitor the data traffic in the entire specified network. Distributed IDSs use multiple IDS agents throughout the network. Centralized IDSs operate from a single monitoring point. In addition to these traditional IDS approaches, current IDS trends in the IIoT include multi-access edge computing (MEC), machine learning integration, lightweight detection frameworks, real-time IDSs, collaborative defense IDS strategies, encrypted traffic IDSs, and blockchain IDSs. MEC works by offloading computational tasks to edge devices. By using machine learning, an IDS can be trained on the expected workflow of a network and detect anomalies with higher accuracy. As IIoT devices can be limited in computing power, lightweight detection frameworks in these edge devices can be useful. Real-time monitoring can minimize damage through instant detection. Through the use of blockchain and collaboration, devices can communicate with each other to jointly detect threats in a temperature-safe manner. Analyzing encryption without breaking the encryption is important in a network with a large number of edge devices, such as an IIoT network [110,111].

### 5.21. Secure Development

In all phases of software development and a product’s lifecycle, security-related activities should be appropriately planned, documented, and executed. The development/maintenance/support process should be documented and enforced. Externally provided components and third-party components must be identified and managed.

IgniteSec, a method specifically for the IIoT, was developed by Filho and Cesar [112]. It integrates security in every stage of IIoT development and is based on the NIST 800-82 standard.

Narayana [113] developed a framework for secure development and audits. It covers audits in every stage of development, from requirements’ definition to maintenance. It describes tools for static analysis, penetration testing, compliance with industry standards such as OWASP, and GDPR compliance.

Shaabany and Anderl [114] demonstrated how the secure development process can be applied to a real-life example of a liquid mixing machine. They integrated security into the requirement identification phase and then conducted a risk assessment. In the design phase, they used techniques to protect against reverse engineering, such as encryption and obfuscation. Secure communication protocols such as TLS were used to encrypt the transmitted data. Digital signatures were used to authenticate the data and storage was secured to protect the data at rest. To secure the product in the production phase, firewalls and antivirus programs were used and audits of the production systems were carried out.

Behrendt et al. [115] emphasize the need for strong supplier management and the selection of suitable partners. This includes the evaluation of partners based on criteria such as scalability, security, and development capabilities. A centralized security framework that manages the integration of third-party vendors is recommended.

### 5.22. Cryptographic Secret Security

Technical and procedural controls must be in place to protect private keys and secrets.

Yu et al. [116] propose STCCHain, which uses the Shamir threshold to split private keys into encrypted fragments that are stored across blockchain nodes. A user must collect enough fragments to reconstruct the key and access the data. Even if some fragments are compromised, the key remains secure. By using this mechanism, both the security of the key and the ability to verify transactions are guaranteed and there is protection against counterfeiting.

R. et al. [104] propose a multi-level authenticated key agreement scheme that uses the Chinese Remainder Theorem (CRT) to create group keys between devices. These group keys help negotiate secure session keys between users and devices. Secrets such as cryptographic keys are protected by hash functions and secret sharing so that malicious actors cannot easily reconstruct them even if individual devices are compromised.

### 5.23. Testing Procedures

The system should be tested to mitigate threats and check for vulnerabilities. The testers should not be the same people as the developers who designed the system. All predefined security functions and capabilities for all IACS components must be tested.

Al-Hawawreh and Sitnikovas [117] provide a test environment that mimics real industrial environments, called the Brown-IIoTbed. It enables security threat analysis using models such as STRIDE, machine learning for intrusion detection, and proactive defense mechanisms such as threat hunting. It is a flexible and scalable testbed that provides the ability to perform end-to-end security testing.

Glock et al. [118] propose a model-based validation approach. It enables the reuse of existing test cases by adapting them to specific systems. It also enables continuous validation throughout the lifecycle of the system, including the planning, configuration, development, and testing phases.

To test critical functions in an IIoT environment, Antão et al. [119] propose using established methods such as hardware-in-the-loop (HiL) testing and adapting them to IIoT environments. Their methods focus on the ability to scale the tests, reuse them, and reduce the cost of testing as well as the need for manual testing.

### 5.24. Decommissioning Procedure

Guidelines for decommissioning a product should be created.

Shirvani and Ghasemshirazi [120] talk about the sustainability of the IIoT. They point out problems such as premature decommissioning, user awareness, and dependence on vendors leading to high levels of electronic waste. Users can be trained to maintain their devices. Devices need to be flexible enough to be upgraded to avoid obsolescence, which should be supported by legal frameworks. When it comes to decommissioning devices, blockchain technology can be used to secure and manage the lifecycle of the device, and smart contracts can be used to automate the decommissioning process.

Rahman et al. [121] provide guidelines for the proper decommissioning of a device. Before decommissioning, the device must be reset to factory settings, testing should be performed to see how decommissioning affects the rest of the system, and then the device must be disconnected from the network.

## 6. Future Directions

Based on the previous chapter, it can be concluded that most of the requirements of IEC 62443 are applicable to the IIoT.

Isolating security-related systems from the rest of the industrial environment has proven difficult in practice. The reason for that is that IIoT systems are interconnected and industrial processes are digitized. This means that the requirements themselves are difficult to implement in any industrial environment.

Some requirements are either so broad that they are applicable to both IACSs and the IIoT or they are researched enough that the research analyzed finds no major problems in securing these aspects of IIoT systems. Those requirements are creating a cybersecurity program, physical security requirements, employee training, creating policies and procedures, defense-in-depth principles, zone segmentation and network segregation and security, wireless communication security, and cryptographic secret protection.

The following requirements are well researched and can be applied to the IIoT but need some adjustments and further research: defining audit methods and procedures, patching procedures, business continuity and testing, identity and authentication management, secure development, media handling, decommissioning, inventory management, risk assessment, change management, data retention, and incident response procedures.

Audit methodologies lack guidance for formal risk assessment across all lifecycle phases. As the number of devices and connections in an IIoT environment is large, the process needs to be formalized, tested, and automated as much as possible. Currently, there is no such tested and proven process for the IIoT. Asset management should be integrated into the audit process. Such integration should provide real-time data that should be automatically updated.

There are no direct guidelines on how to check whether the patching process of an IIoT device affects the essential functions of the IACS. There is also no framework on how to document the patch information in an IIoT system according to the standard. A specific patching procedure for the IIoT should be created. It should show how to test the network, the entire system, and all geographically distributed IIoT devices that are part of the system.

In terms of business continuity, these papers do not define specific recovery objectives and the impact of system failure in detail. There is no mention of how to define the roles for such a process and what the key actors are in the context of the IIoT. There is no discussion of how backup and recovery procedures are integrated into a larger business continuity plan. The key actors of the IIoT system and what their role is in a larger business continuity process need to be defined.

Identity and authentication are comprehensive requirements that need to be formalized for the IIoT. The papers analyzed do not mention how all connections to all devices are defined, either for machine-to-machine or human-to-machine communication, so that the purpose, type, location, identity, and duration are always known. The regular checking of authorizations has not yet been sufficiently researched in an IIoT environment. Session length control for IIoT devices is also underexplored. The monitoring and control of network requests for an IIoT environment is not covered in enough detail in these papers.

When it comes to secure development, there is not enough research on whether the development process for the IIoT needs to follow some IIoT-specific requirements that are not applicable to general IACSs. That is, there is not enough research on the differences between IACSs and the IIoT when it comes to developing applications and devices. Although the research analyzed provides a solid foundation for integrating security into each phase of development, there is a lack of comprehensive guidelines for security-related activities throughout a product’s lifecycle.

Research on media handling is limited and no specific solution has been found for many parts of the requirements. A framework for access policies and the handling of IIoT devices needs to be created. There is no reference to the labeling, handling, storage, and transportation of media in the context of the IIoT. Some devices require physical access and updates via physical media.

Decommissioning procedures lack a framework for dealing with a large number of devices spread over a large area and potentially inaccessible. The papers provide a good general overview of safe and responsible decommissioning, such as resetting devices and the secure handling of cryptographic keys. However, they lack concrete operational procedures that should be documented step by step and enforced as part of a secure lifecycle management strategy.

The inventory process lacks a system for maintaining an up-to-date and accurate list of software versions and retention periods. In addition, the method for defining the links between roles and available assets is unclear.

The methods for risk assessment for IIoT are sufficiently researched, apart from clear guidelines for a regular reassessment of risks throughout the lifecycle of an IIoT system, which is a key requirement of the IEC 62443 standard.

Research on change management lacks guidelines on how to document, track, and maintain all assets in a timely manner. None of the authors provide specific tools or methods to comprehensively address change management in an IIoT context. There is also limited discussion of how to ensure the documentation, tracking, and timely maintenance of all assets. Additionally, there is a lack of detailed security guidelines to govern changes in IACS components.

There is a lack of discussion on data retention and deletion policies, particularly cryptographic controls and data stream protection. The documentation of monitored access points is also under-researched, as are security requirements for device-to-device communication.

There is not enough IIoT-specific incident response research. Specific recovery objectives and the impact of system failure are not defined in detail.

Future research could focus on further specifying any of the requirements which were found not to be fully applicable, as described above.

Hardening procedures for the IIoT have not been adequately researched as the scope of this requirement is very large. No comprehensive guide or framework has been found on how to fully harden an IIoT environment.

### Hardening in an IIoT Environment

Echeverría et al. [122] provided guidelines for IoT hardening, but the paper did not address specific requirements for IIoT hardening, nor did it intend to do so. The authors conducted an extensive literature review and found a lack of comprehensive hardening solutions specifically tailored to the IoT, and they proposed a framework for IoT hardening.

However, the IIoT has its own operational requirements and constraints.

One important area not covered is the high availability requirements specific to industrial systems. Unlike general IoT systems, IIoT systems require robust redundancy planning and advanced fault detection mechanisms, usually implemented through dynamic asset management and real-time monitoring.

The methodology presented in the discussed paper focuses on the use of modern IoT devices, leaving a gap in dealing with legacy devices that are often unavoidable in IIoT contexts. Legacy systems may not have built-in security features and are frequently proprietary. A suitable IIoT hardening process would therefore need to include strategies to securely integrate these older devices through retrofitting.

Another key difference between the IoT and IIoT lies in the specific communication protocols used in industrial environments. These protocols differ from those used in consumer IoT systems and require specialized security strategies to address their specific vulnerabilities.

They also lack the hardening of safety controls, an essential component of IIoT security design. IIoT systems control physical processes, and security failures can have serious physical consequences. Therefore, an appropriate hardening model should include steps to ensure physical security.

Initial research on this topic has already been carried out by Laszka et al. [107]. They propose a framework that, among other things, discusses the hardening process from the point of view of cost-efficiency. They mention the need for multiple layers of defense. To optimize security investments, the paper proposes a model to quantify risks and an efficient meta-heuristic algorithm to find near-optimal security designs. However, the main focus of the paper is on the efficiency of security investments and not on the design and problems of creating an all-encompassing hardening framework.

However, the areas that were found to be critical when analyzing the IIoT through the requirements defined in IEC 62443 have not been adequately addressed in any paper dealing with the hardening of IIoT systems.

## 7. Conclusions

The mapping of IIoT requirements to the ISA/IEC 62443 standards shows that many of the cybersecurity requirements for industrial automation and control systems (IACSs) can be applied to IIoT environments, but there are still significant gaps in adapting many of these requirements to an IIoT environment.

These gaps exist due to the unique nature of the IIoT. These devices have low power consumption, their numbers are far greater than the number of devices in a standard IACS environment, and they rely on cloud computing, edge computing, or a combination of such technologies, and integration with AI technologies is far more common than it was in the past. All this means that the IIoT brings with it a host of new use cases, scenarios, vulnerabilities, threats, and risks that were previously unknown in IACS environments.

As no specific standard for the IIoT exists to date, the research gaps identified in this paper are a result of the fact that the IIoT has not been considered on the basis of a standard, meaning that a formalization of many of the processes, systems, and policies required for ISA/IEC 62443 was never created for the IIoT.

For this reason, a broader standard must be created that also covers the IIoT, or ISA/IEC 62443 needs to be adapted to also take into account the specifics of the IIoT.

One of the most critical areas that requires further research is the hardening of IIoT systems.

Given the complexity and dynamic nature of the IIoT, especially with the integration of devices with limited resources, robust hardening techniques are essential for securing these systems. Current research offers some insights into various aspects of IIoT security, but there is a notable lack of comprehensive frameworks or guidelines for the hardening process that could be considered formal enough and broad enough to be included in a new IIoT-applicable standard.

Future research should include the development of detailed, formal, standardized, IIoT-based procedures for audit methods, patching procedures, business continuity plans, identity and authentication management, secure development, media handling, decommissioning, inventory management, risk assessment, change management systems, data retention policies, incident response, and especially hardening.

## Data Availability

Data are contained within the article.
